# The Interplay Among HIV, LINE-1, and the Interferon Signaling System

**DOI:** 10.3389/fimmu.2021.732775

**Published:** 2021-09-09

**Authors:** Xu Zhao, Yifei Zhao, Juan Du, Pujun Gao, Ke Zhao

**Affiliations:** ^1^Institute of Virology and AIDS Research, First Hospital of Jilin University, Changchun, China; ^2^Department of Hepatology, First Hospital of Jilin University, Changchun, China; ^3^Key Laboratory of Organ Regeneration & Transplantation of the Ministry of Education, First Hospital of Jilin University, Changchun, China

**Keywords:** human immunodeficiency viruses, type 1 long interspersed elements, IFN signaling system, sensors, interferon-stimulated genes, restriction factors, autoimmunity

## Abstract

Human immunodeficiency viruses (HIVs) are retroviruses that replicate effectively in human CD4^+^ cells and cause the development of acquired immune deficiency syndrome (AIDS). On the other hand, type 1 long interspersed elements (LINE-1s or L1s) are the only active retroelements that can replicate autonomously in human cells. They, along with other active yet nonautonomous retroelements, have been associated with autoimmune diseases. There are many similarities between HIV and LINE-1. Being derived (or evolved) from ancient retroviruses, both HIV and LINE-1 replicate through a process termed reverse transcription, activate endogenous DNA and RNA sensors, trigger innate immune activation to promote interferon (IFN) expression, and are suppressed by protein products of interferon-stimulated genes (ISGs). However, these similarities make it difficult to decipher or even speculate the relationship between HIV and LINE-1, especially regarding the involvement of the IFN signaling system. In this review, we summarize previous findings on the relationships between HIV and innate immune activation as well as between LINE-1 and IFN upregulation. We also attempt to elucidate the interplay among HIV, LINE-1, and the IFN signaling system in hopes of guiding future research directions for viral suppression and immune regulation.

## Introduction

Being a part of the innate immune system, the interferon (IFN) signaling system is a critical defense for a host (such as humans) to prevent viral infection and replication (1). The IFN signaling system can be roughly divided into three parts. The first part contains many receptors and several pathways activated by them (2). These receptors recognize ligands with specific patterns and hence are termed pattern recognition receptors (PRRs). Certain components (such as DNA/RNA or proteins) from viruses can interact with PRRs and initiate the activation of the host’s antiviral defense. The second part comprises enigmatic proteins called interferons (IFNs). Upon the activation of upstream pathways initiated by PRRs, the levels of IFNs are increased (3). These IFNs, however, have not been found to have viral suppression activity by themselves. Instead, they promote the expression of interferon-stimulated genes (ISGs) (1), which constitute the third and final part of the IFN signaling system. Many protein products of ISGs are effective against viruses (4–6); these antiviral proteins restrict viral ability to infect and/or replicate and thus are sometimes called “restriction factors”.

Ideally, in the name of benefiting the host, the IFN signaling system should sense the presence of viruses, suppress viral infection/replication, and keep the host healthy. However, humans can sometimes develop diseases triggered by an overactivated IFN signaling system without the detection of foreign viruses or other pathogens (7). The development of these autoimmune diseases indicates the existence of endogenous triggers of the system (i.e., endogenous ligands of PRRs), and increasing evidence has suggested that some of these triggers are retroelements (8, 9). Intriguingly, in human cells, the only active type of retroelements that can replicate autonomously are type 1 long interspersed elements (LINE-1s or L1s), which also support the retrotransposition of other active yet nonautonomous retroelements, such as Alu and SVA (10). Accordingly, revealing how LINE-1 triggers innate immune activation has attracted increasing attention because it might uncover the mechanism of autoimmunity and possibly lead to the control or even cure of autoimmune diseases.

**Figure 1:**
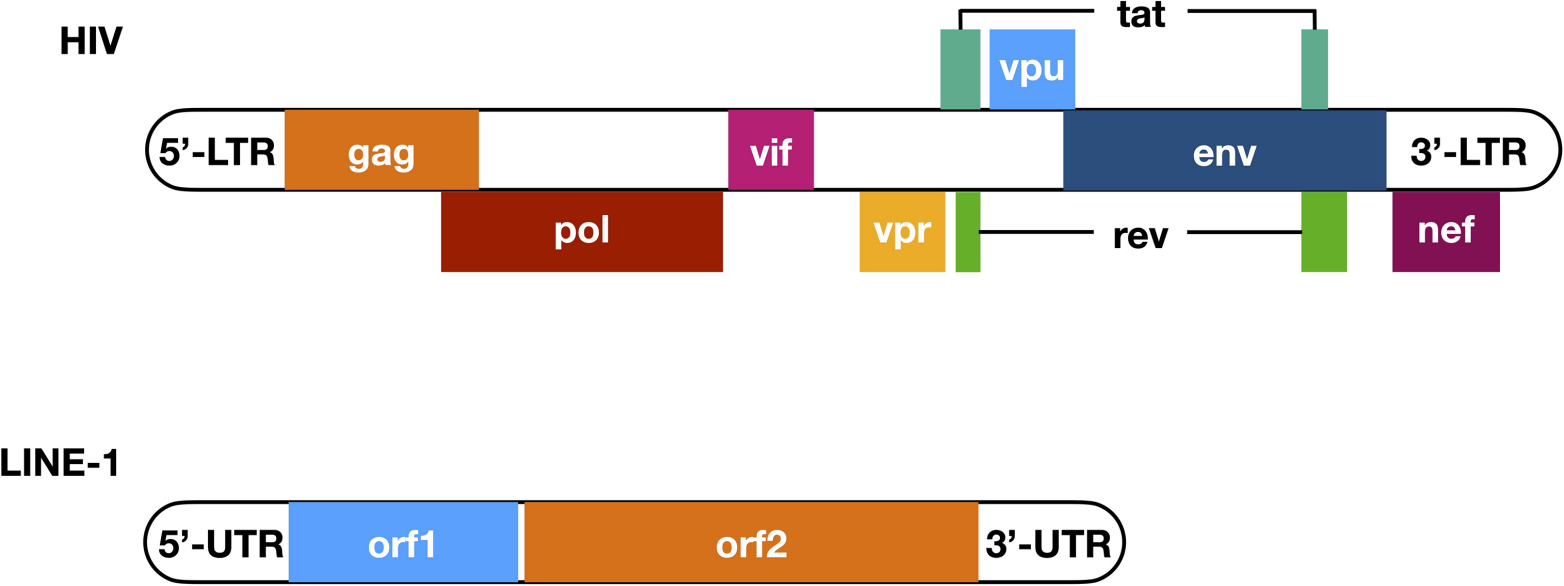
Primary structure of HIV genome and LINE-1 element. Nine genes have been determined in the HIV genomic RNA (with overlapped genes placed next to the main sequence of HIV genome), among which three encode structural proteins Gag, Pol, and Env, two encode regulatory proteins Tat and Rev, and four encode accessory proteins Vif, Vpr, Vpu, and Nef. Notably, *tat* and *rev* are the only two viral genes that have two exons. These nine genes are flanked with 5’- and 3’-LTR, both of which are essential for HIV replication, with the 5’-LTR contains a promoter sequence. On the other hand, a LINE-1 element has a 5’-UTR that functions as a promoter and ribosome binding site in its DNA and RNA form, respectively, and a 3’-UTR that enhances the efficiency of LINE-1 retrotransposition. In between, there are two open reading frames named *orf1* and *orf2*, while the encoded ORF1p and ORF2p interact with LINE-1 RNA and provide endonuclease and reverse transcriptase activities that are critical for LINE-1 retrotransposition. LTR, long terminal repeats; UTR, un-translated region.

On the other hand, while endogenous retroelements are considered artifacts of ancient retroviruses (11), modern retroviruses such as human immunodeficiency viruses (HIVs) transmit among humans and cause the development of acquired immune deficiency syndrome (AIDS) (12, 13). As a species of the *Retroviridae* family, HIV has an RNA genome, which consists of ~9.4k nucleotides on average. Flanked with both 5’ and 3’ long terminal repeats (LTR), nine genes have been determined in between (**Figure 1**). The encoded viral proteins can be divided into three categories. Gag, Pol, and Env as structural proteins are essential for the assembly of new HIV virions (14). Tat and Rev as regulatory proteins regulate the transcription and processing of HIV RNA and are indispensable for effective HIV replication (15–19). Vif, Vpr, Vpu (Vpx in HIV-2), and Nef were once considered as “accessory proteins” because they are not strictly required for HIV infection in certain cell lines (20–22). But now it has been widely accepted that these proteins are essential for both viral infectivity and pathogenesis *in vivo*, mostly because their ability to counteract host innate immune defense (23–33).

Similar to that of LINE-1, the replication of HIV also includes reverse transcription, meaning that HIV infection introduces viral RNA and DNA into human cells. These exogenous RNA and DNA are perfect targets for endogenous sensors to recognize, and the resulted innate immune activation leads to the elevation of IFNs and subsequently the expression of ISGs. Many products of ISGs, such as the APOBEC3 protein family (34), TRIM5α (35), and MX2 (36–38), have been reported to suppress or even terminate HIV replication. However, to optimally replicate, HIV with its encoded proteins has also evolved multiple counteraction mechanisms to compromise these ISGs, such as inducing their degradation (27, 29, 30, 39), altering their subcellular localization (40), or even compromising upstream pathways to prevent innate immune activation (41). Apparently, LINE-1 functions as an endogenous trigger of the IFN signaling system, producing a dilemma for HIV: because LINE-1 originates from ancient retroviruses and thus shares similarities with HIV, the presence of HIV or its component(s) might enhance LINE-1 activity, which could lead to innate immune activation that is inhibitory for HIV replication; alternatively, HIV must have evolved and possess alternative mechanism(s) so that the same viral protein can simultaneously function as an HIV promoter and LINE-1 suppressor. Therefore, summarizing previous findings is of importance for future studies revealing the relationship among HIV, LINE-1, and the IFN signaling system.

## HIV and the IFN Signaling System

### The IFN Signaling System Senses HIV Components

**Figure 2:**
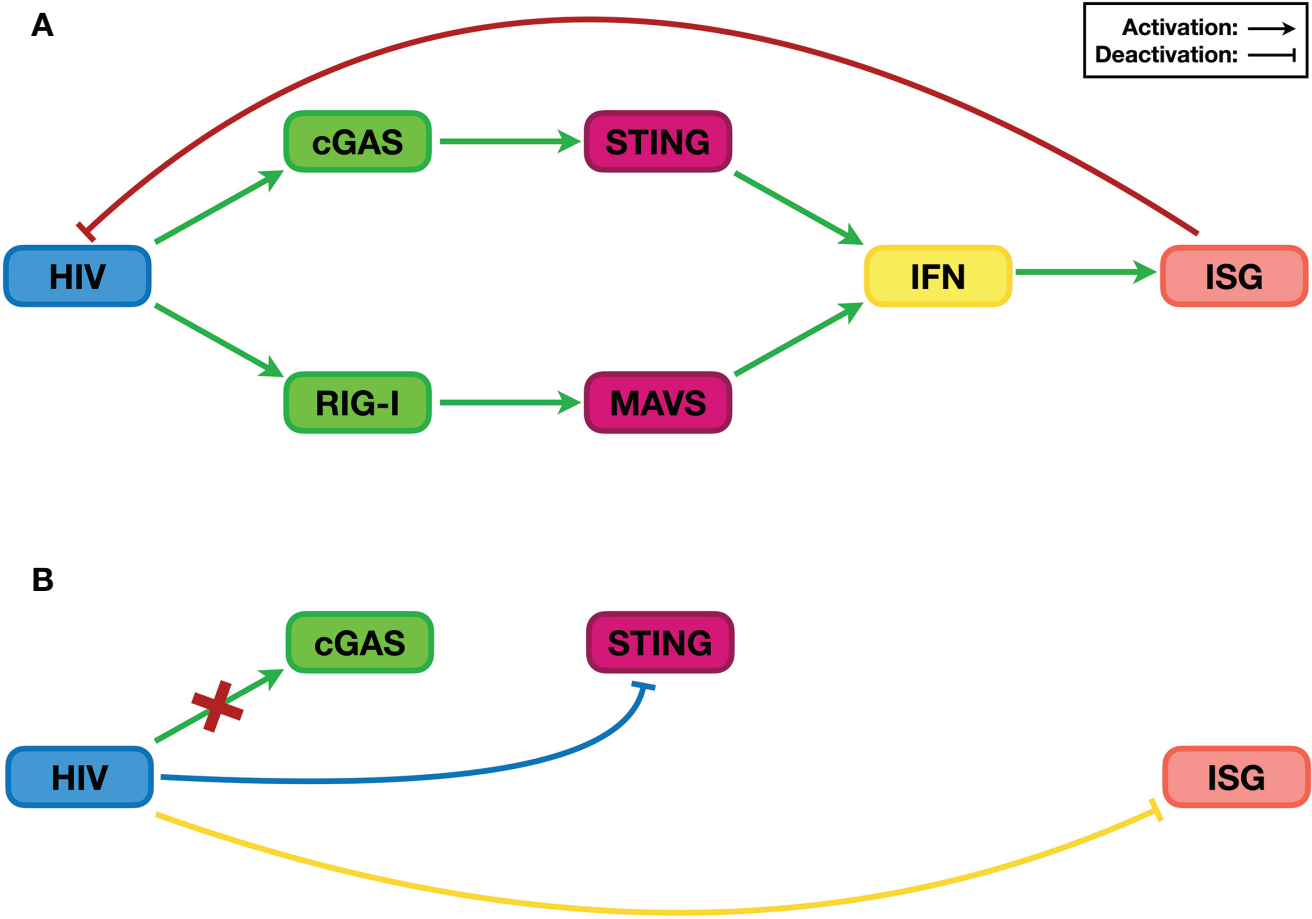
The relationship between HIV and the IFN signaling system. Post infection, HIV RNA and its reverse-transcribed cDNA can be recognized by the endogenous RNA sensor RIG-I and DNA sensor cGAS, respectively, which then trigger innate immune activation and induce the expression of ISGs [panel **(A)**, green pathways]. Many ISG proteins are restriction factors that suppress HIV infection [panel **(A)**, red pathway]. As a counteraction, HIV evades the sensing by the DNA sensor cGAS [panel **(B)**, red cross], suppresses the activation of the upstream sensing pathway(s) [panel **(B)**, blue pathway], and compromises the stability/functionality of ISG proteins [panel **(B)**, yellow pathway] to ensure viral replication.

The RNA nature of HIV genome suggests that it could be recognized by human proteins that sense the presence of exogenous RNA from either viral genomes or transcripts. MDA5 and RIG-I are the two most important RNA sensors in human cells, with MDA5 preferably recognizing double-stranded RNA (dsRNA) and RIG-I sensing uncapped long single-stranded RNA (ssRNA) (42). In the case of HIV, this interplay is somewhat intricate. Viral ssRNA is >9 kb on average but capped and polyadenylated at the 5’ and 3’ ends, respectively, the same as host mRNAs. On the other hand, it also contains multiple secondary structures in the 5’- and 3’-untranslated regions, which may be recognized as patterns by RNA sensors. As a result, RIG-I but not MDA5 senses the HIV genome (43) (**Figure 2A**, green pathway). Indeed, the presence of HIV RNA not only increases the expression of IFNβ but also elevates the protein levels of many ISGs, such as ISG15, ISG56, APOBEC3G, and CXCL10. However, the details regarding HIV RNA activation of RIG-I remain mostly unknown. Among these tested ISGs, APOBEC3G is widely known for suppressing HIV infectivity by introducing “G-to-A” mutations in the viral genome through its deaminase activity (34), while ISG15 deficiency was recently found to promote HIV infection (44); there are additional anti-HIV factors whose levels might also be elevated. Consequently, RIG-I-mediated IFN production in the presence of the HIV RNA genome effectively reduces HIV replication (43) (**Figure 2A**, red pathway).

Similar to other retroviruses, HIV also includes reverse transcription in its replication process, leading to the generation of viral complementary DNA (cDNA), which in turn triggers innate immune activation. Interestingly, the latter step was not uncovered by a direct study on the relationship between free (rather than integrated) HIV DNA and the IFN signaling system but rather through a search for an antiviral factor (45). Three-prime repair exonuclease 1 (TREX1) is an endogenous nuclease digesting linearized ssDNA and dsDNA (46). Since reverse-transcribed HIV cDNA is ssDNA, it was easy to speculate that TREX1 might suppress HIV by decreasing the levels of HIV reverse transcripts. The test results, however, indicated that although TREX1 indeed reduces HIV cDNA levels, it increases HIV replication. Soon, it was determined that by digesting HIV cDNA, TREX1 helps HIV prevent the activation of the IFN signaling system, and the combined outcome is promotion of HIV infection (45). Nevertheless, three years later, researchers finally uncovered the endogenous DNA sensor cyclic GMP-AMP synthase (cGAS), which is essential for HIV cDNA recognition (47). Upon binding HIV cDNA, cGAS synthesizes cyclic GMP-AMP (cGAMP), which functions as a secondary messenger to activate the STING and subsequent IRF3 and NF-κB pathways and ultimately triggers the expression of IFNs, among many other proteins (48, 49) (**Figure 2A**, green pathway). Therefore, during its replication process, HIV activates the IFN signaling system through both RNA- and DNA-sensing pathways, which correlates with the sensing role of the RNA sensor RIG-I or the DNA sensor cGAS.

### HIV Can Compromise, Suppress and Evade the IFN Signaling System

**Figure 3:**
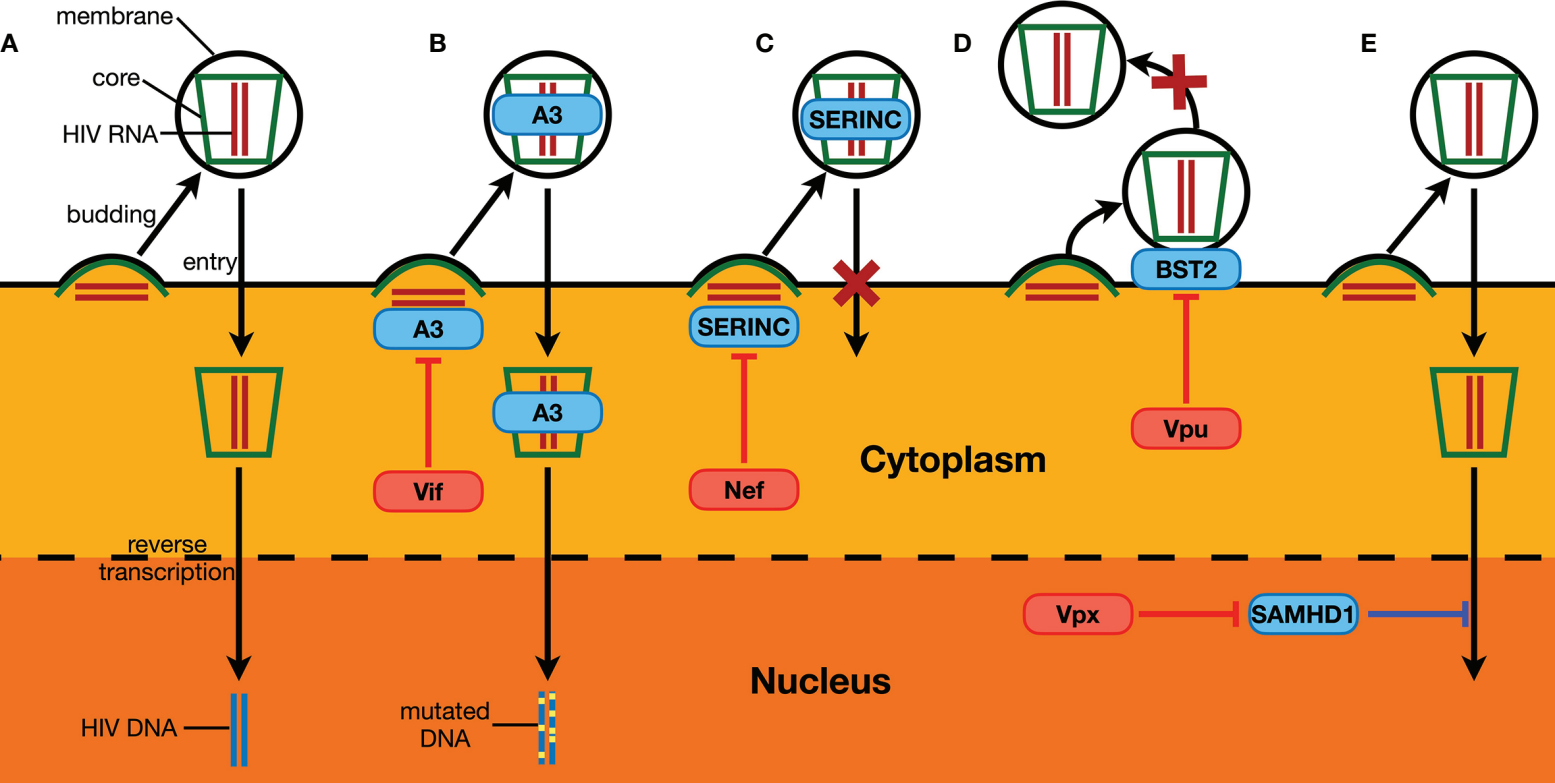
Examples of host restriction factors suppressing HIV replication and associated viral counteraction. **(A)** As a part of HIV replication process, synthesized HIV RNA and proteins trigger the budding of a new HIV particle, which ultimately releases a virion with a central core structure containing two HIV genomic RNA and being covered with a cell-derived membrane. The virion then recognizes another target cell, and the core structure enters through membrane fusion between the host cell and the virus. This is followed by the reverse transcription of HIV RNA, and the synthesized viral DNA is then injected into the nucleus, ready to be integrated in the host genome. **(B)** Members of APOBEC3 proteins (A3) can be packaged into HIV virions. During the next-round infection, APOBEC3 proteins can introduce “G-to-A” mutations to HIV DNA through its deaminase activity, which ultimately leads to the loss of infectivity of newly produced virions. As a counteraction manner, HIV Vif protein induces the depletion of APOBEC3 proteins by hijacking host proteasome-mediated proteolysis. **(C)** Similarly, SERINC proteins can also be packaged into HIV virions. When encountering new target cells, HIV virions containing SERINC proteins can no longer trigger the membrane fusion between the host cell and the virus, through which the entry of the viral core structure is sabotaged. The Nef protein expressed from HIV genome can interact with SERINC proteins and prevent the latter from being packaged into HIV virions. **(D)** BST2 is a restriction factor that is normally localized on membrane structures of a host cell, including the cytoplasmic membrane where the budding of HIV virions happens. The protein can insert its one end into the viral membrane during the budding process of HIV, and keeps the other end stay on the cytoplasmic membrane. Consequently, BST2 forms a physical link between the assembled HIV virion and the host cell, which prevents the viral particle from being released. Such an antiviral mechanism could be counteracted by HIV Vpu, which removes BST2 from the cell surface. **(E)** SAMHD1 is the only known dNTPase in human cells. It potently reduces HIV infectivity in non-dividing cells such as macrophages and dendritic cells, by compromising viral reverse transcription process and lowering the synthesis of HIV DNA. Vpx that is specifically expressed by HIV-2 removes SAMHD1 through host proteasome-mediated proteolysis.

The examples above have shown that the activation of the IFN signaling system, through RNA- and/or DNA-sensing pathways, compromises HIV replication. Based on current knowledge, to achieve optimal replication, HIV utilizes three general methods to counteract host innate immune activation. The first is to reduce the levels and/or functions of ISG proteins (**Figure 2B**, yellow pathway). The degradation of the restriction factor APOBEC3G by the HIV Vif protein is the most famous and well-studied case. Soon after the discovery of HIV as the causative reagent of AIDS, researchers found a viral protein that is essential for viral replication in certain types of cell lines; accordingly, this viral protein was named viral infectivity factor (Vif) (22). However, the mechanism was not revealed until 2003 (25–27). When expressed in host cells, Vif interacts with host proteins such as Cullin5, Elongin B, and Elongin C, which are key components of a ubiquitin ligase complex (or E3 complex) (27). There are many kinds of E3 complexes in human cells, and each mediates ubiquitination of specific host proteins that are then degraded through proteasome-mediated proteolysis. By hijacking the Cullin5-based E3 complex and binding to APOBEC3G, Vif forces the ubiquitination and subsequent degradation of APOBEC3G (25, 26). As mentioned above, APOBEC3G can introduce mutations into the HIV genome, resulting in suppressed viral infectivity (50). By reducing APOBEC3G levels, Vif protects the HIV genome, maintains viral infectivity, and thus enhances HIV replication (**Figure 3B**).

In addition to Vif-induced APOBEC3G degradation, there are other examples of a viral protein counteracting the stability and/or function of ISG proteins (**Figure 3**). For instance, members of the SERINC family such as SERINC 3 and SERINC 5 can also be packaged into HIV virions and prevent membrane fusion between the virus and the targeted cell (51, 52). Nef expressed from some HIV strains can reduce endogenous levels of these SERINC proteins, which promotes the infectivity of newly produced HIV particles (31, 32) (**Figure 3C**). BST2 as another ISG protein physically tethers newly produced HIV virions and prevents them from being released, and thus is also named “tetherin” (53). HIV Vpu removes host BST2 protein from the surface of infected cells, which is essential for HIV virion to release and initiate the next-round infection (54, 55) (**Figure 3D**). In addition, SAMHD1 is a potent HIV suppressor in non-dividing cells such as macrophages and dendritic cells (29, 30). It inhibits HIV reverse transcription, where its dNTPase activity is believed essential (56, 57). As a counteraction manner, Vpx from HIV-2 induces the depletion of the host SAMHD1 protein through proteasome-mediated proteolysis, which is critical for HIV-2 to infect and replicate in macrophages (29, 30) (**Figure 3E**). Intriguingly, in addition to sensing HIV RNA, RIG-I is also an ISG protein and can potently suppress HIV replication by itself, which can be counteracted by the presence of viral protease (43).

The second manner by which HIV sabotages the IFN signaling system is by actively compromising the efficiency of upstream sensing pathways (**Figure 2B**, blue pathway), which is relatively less studied. Recently, Su et al. (41) noticed that although the Vpx from HIV-2 or simian immunodeficiency virus (SIV) could induce the depletion of endogenous SAMHD1, which causes the accumulation of DNA fragments in the cytoplasm, infection of wild-type viruses did not trigger elevated levels of innate immune activation compared to those triggered by viruses lacking the *vpx* gene. Inspired by this phenomenon, they discovered that the viral Vpx protein interacts with STING, an essential component of the endogenous DNA-sensing pathway, and suppresses the latter’s ability to trigger downstream innate immune activation. Interestingly, this pathway is different from Vpx inducing SAMHD1 depletion through proteasome-mediated proteolysis; instead, the interaction between Vpx and STING is both necessary and sufficient for the inhibition of STING function. Thus, through STING regulation, Vpx protects viruses from suffering the antiviral consequence of removing SAMHD1 and maintains the efficiency of viral replication.

The above research on HIV and TREX1 provides a perfect example on the third approach that HIV can evade the IFN singling system (**Figure 2B**, red cross). TREX1 as a DNA exonuclease reduces levels of HIV reverse transcripts, which should subsequently affect viral integration; in this point of view, TREX1 should be considered as a restriction factor to suppress HIV infection. However, HIV does not try to compromise the function/stability of TREX1 as it does to many other restriction factors as mentioned above. Instead, it passively endures the exonuclease activity of TREX1, through which the virus prevents the activation of DNA-sensing pathways and enhances viral replication (45).

It is difficult to determine which manner is better for HIV to sabotage the IFN signaling system, as each has its advantages and disadvantages. For example, targeting restriction factors can compromise host antiviral defense in a shorter period because it directly removes these restriction factors and/or suppresses their antiviral functions, leading to innate immune suppression without involving upstream sensing pathways. Additionally, it can occur as soon as the virus enters the cell, which, for instance, is the time when packed Vpx starts to remove SAMHD1 in target cells (58). However, it also requires the virus to recognize these many restriction factors with limited numbers of viral proteins, and sometimes one viral protein needs to target multiple factors, such as the fact that one Vif protein induces the degradation of several APOBEC3 proteins and PPP2R5 (59, 60). This requirement can lock the sequence of viral proteins and limit their capability of further evolution. On the other hand, by compromising endogenous sensing pathways, one viral protein can cause a decrease in the levels of hundreds or even thousands of ISG proteins, thus supporting viral replication in a wider spectrum. However, this is a slower process because the final result of compromising sensing pathways is the reduction in newly synthesized ISG transcripts, while ISG proteins that have been expressed before infection still need time to decay. During the same period, HIV infection might have already been terminated due to suppressed reverse transcription, harmful genome mutagenesis, etc. Similar advantages and disadvantages also apply for HIV to evade the IFN singling system, with additional drawbacks like the reduction of viral component(s) as observed with TREX1 (45). Therefore, since neither targeting restriction factors nor compromising/evading sensing pathways can fulfill the needs of HIV to maintain its infectivity, it is reasonable that HIV utilizes all three to promote viral infection/replication at any given time and reduce the levels of restriction factors as much as possible. However, a question remains: if there were endogenous triggers whose presence and/or activity could activate the IFN signaling system, would HIV have evolved another manner to regulate IFN expression by suppressing this trigger?

## LINE-1 and the IFN Signaling System

### The Study of Aicardi-Goutières Syndrome Links LINE-1 to Autoimmunity

To answer the above question, we first need to know the nature of endogenous triggers of innate immune activation, the knowledge of which mostly has come from the study of autoimmune diseases. In the early 1980s, a rare autoimmune disease was characterized, with the most significant phenomenon being an increase in IFNα levels in cerebral-spinal fluid (61). The full characterization of this disease was first described by Aicardi and Goutières, and thus, the disease was later named Aicardi-Goutières syndrome (AGS). AGS was soon found to be an autosomal recessive disorder, and several genes that are associated with AGS have been identified since [refer to (62) for additional details]. The first AGS-associated gene identified is *TREX1* (63), and the gene product TREX1 has long been known for its activity as an exonuclease that digests linearized DNA (46). Therefore, it seemed reasonable to suspect that TREX1 prevents innate immune activation through its exonuclease activity. Consistently, two subsequently published studies confirmed that eliminating TREX1 expression in mice could induce phenomena that mimicked autoimmunity (64, 65). Specifically, one of these two studies revealed that in the heart tissue of *TREX1* knockout mice, an increase in linearized DNA levels could be detected (65). Intriguingly, such an increase was not apparent for all kinds of DNA; instead, the elevation was strictly observed for DNA fragments derived from retroelements, including LINE-1 itself and LINE-1-supported retroelements, such as Alu and SVA. This finding is the first clear evidence that retroelements might be a potential trigger of innate immune activation.

The hypothesis that LINE-1 induces IFN production was further supported by subsequent studies. Beginning with *TREX1*, additional AGS-associated genes were unveiled (66–69). All AGS-associated proteins are linked to LINE-1 activity. TREX1, SAMHD1, and ADAR1 have been reported with clear evidence indicating their potency against LINE-1 retrotransposition (65, 70, 71), while despite controversy regarding its role in support either or suppression, RNase H2 has also been linked to LINE-1 activity (72, 73). Most importantly, similar to the case with TREX1 (65), AGS-associated mutations (both point mutations and truncations) have also been shown to significantly compromise SAMHD1’s potency in LINE-1 suppression (70). Therefore, it appears that LINE-1 is indeed associated with innate immune activation.

### LINE-1 Triggers Innate Immune Activation Through Both DNA- and RNA-Sensing Pathways

**Figure 4:**
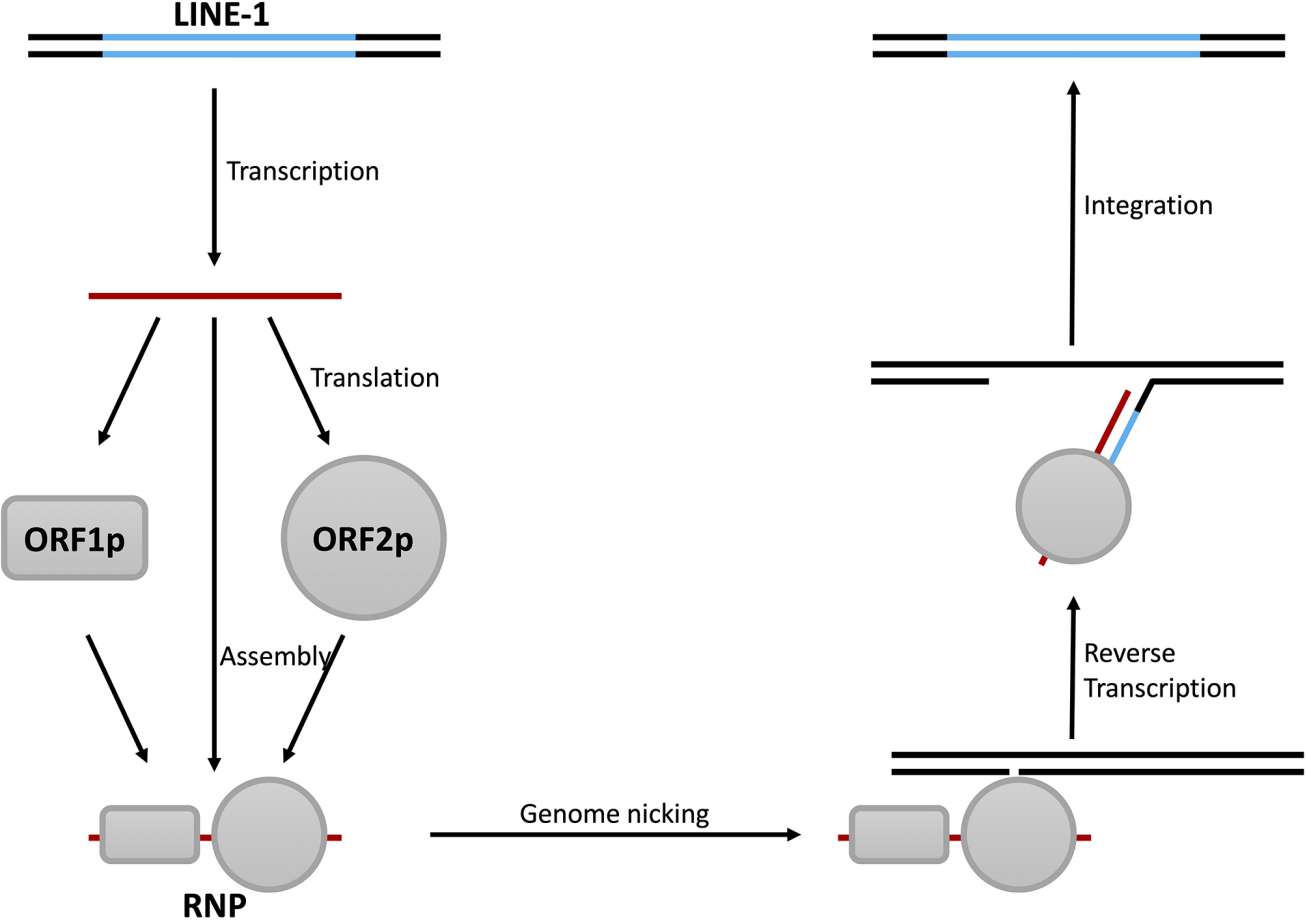
Steps of LINE-1 retrotransposition. The replication of LINE-1 starts with the transcription of LINE-1 RNA and is followed by the synthesis of the LINE-1 proteins ORF1p and ORF2p. ORF1p, ORF2p, and LINE-1 RNA then form LINE-1 RNPs with the help of other cellular proteins. LINE-1 RNPs then bind to genomic DNA and induce nicks with the endonuclease activity of ORF2p. This results in the unwinding of the DNA and leaves a protruding single-stranded fragment as the primer, and LINE-1 cDNA is synthesized with the reverse transcriptase activity of ORF2p. Finally, through a series of unknown mechanism(s), the double-stranded form of LINE-1 DNA is synthesized and integrated into the genome.

Although several phenomena above link LINE-1 to IFN production, direct evidence is still missing. Clearly, revealing how LINE-1 promotes IFN expression would solve this problem. To do that, we need to first understand the biology of LINE-1. LINE-1 was first found in partial in a DNA library generated from the spleen tissue of a female patient homozygous for β^+^ thalassemia (74), while the full-length version was later determined based on a monkey genomic library (75). A typical LINE-1 fragment is 6 kb in length, containing two open reading frames termed *orf1* and *orf2* (11) (**Figure 1**). The encoded ORF1p interacts with LINE-1 RNA and initiates the formation of LINE-1 ribonucleoprotein particles (RNPs), which are the fundamental units for LINE-1 retrotransposition (76). ORF2p also interacts with LINE-1 RNA and is another essential component of LINE-1 RNP (77) because it provides endonuclease and reverse transcriptase activities that are critical for LINE-1 replication (78, 79). During LINE-1 replication, endonuclease activity introduces nicking at the insertion site, and the reverse transcriptase drives LINE-1 cDNA synthesis (11) (**Figure 4**). Recent studies also revealed that there is an antisense coding frame, *orf0*, on the double-stranded LINE-1 DNA fragment, and the coded ORF0p also elevates LINE-1 replication efficiency in a yet-unknown mechanism (80).

**Figure 5:**
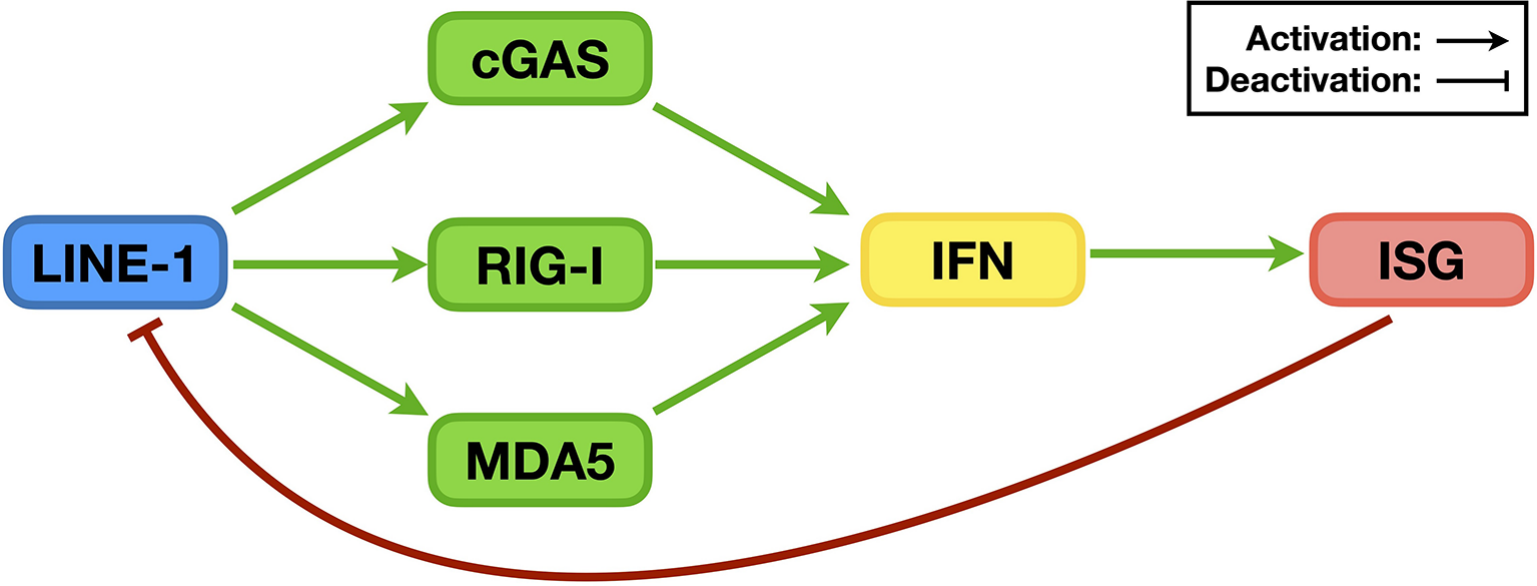
The relationship between LINE-1 and the IFN signaling system. During replication, LINE-1 triggers the activation of the DNA sensor cGAS and RNA sensors RIG-I and MDA5, promotes IFN levels, and enhances the expression of ISGs (green pathways). On the other hand, many ISG proteins are potent LINE-1 regulators that suppress LINE-1 retrotransposition (red pathway).

As mentioned above, the early study showing that TREX1 reduces endogenous levels of LINE-1 DNA provided the first clue that LINE-1 might trigger IFN production through the DNA-sensing pathway(s). However, no further information could be retrieved because the associated DNA sensor was not found until five years later. In 2013, it was finally discovered that cGAS is an important DNA sensor in human cells (48). Soon, it was determined that the presence of cGAS is critical for the innate immune activation observed in *TREX1* knockout cells or even mice (81, 82). In addition, during replication, LINE-1 introduces nicking and causes damage or even breaks to the host genome (83, 84), while damaged genomic DNA is also a target for cGAS recognition (85). Taken together, these results suggest that LINE-1 can promote IFN production through the cGAS-mediated DNA-sensing pathway (**Figure 5**, green pathway). Consistently, increasing evidence confirms the association between elevated LINE-1 DNA levels and promoted IFN expression in studies based on different models or autoimmune diseases (86–88).

It is thus easy to speculate that if DNA sensing is the only mechanism, compromising LINE-1 reverse transcription or destabilizing LINE-1 DNA would be sufficient to suppress LINE-1-induced innate immune activation. However, to date, there is still no direct evidence confirming that LINE-1 DNA interacts with cGAS and triggers innate immune activation. Even indirect tests sometimes give controversial results, and the use of nucleoside reverse transcriptase inhibitors (NRTIs) is a perfect example. Activated NRTIs are a type of small molecule with structures similar to those of dNTPs. Consequently, they block reverse transcription through a competitive mechanism and thus are used as therapeutics for HIV infection (89–91). Through a similar mechanism, they also suppress LINE-1-mediated retrotransposition, which is supported by the reverse transcriptase activity of ORF2p (92). Theoretically, NRTI treatment should inhibit the generation of LINE-1 cDNA and lead to suppressive effects against innate immune activation. However, different results have been observed in different studies conducted by different groups. Truvada [a mixture of two NRTIs termed emtricitabine (FTC) and tenofovir disoproxil fumarate (TDF)] was shown to be effective in suppressing the murine IFN signaling system if used in combination with Viramune [containing nevirapine (NVP) as a nonnucleoside reverse transcriptase inhibitor or NNRTI] (93). A more recent study demonstrated that NRTIs possess intrinsic anti-inflammatory activity, with the use of stavudine (d4T, another NRTI) leading to reduced levels of IFNγ in murine serum (94). As LINE-1 (or the ORF2p protein) is the only known source for endogenous reverse transcription activity, these data seem to correlate with the hypothesis that LINE-1 triggers innate immune activation through DNA-sensing pathway(s). However, when TREX1-mediated LINE-1 suppression was first linked to the development of autoimmune diseases, azidothymidine (AZT, an NRTI that is widely used in HIV treatment) was tested and found to not ameliorate the inflammatory symptoms detected in *TREX1* knockout mice (65). This finding means that sometimes suppressing reverse transcription alone might not be sufficient for proper shutdown of LINE-1-induced innate immune activation; in other words, DNA sensing might not be the only pathway by which LINE-1 activates the IFN signaling system.

A similar doubt was also raised by unveiled mechanisms of LINE-1 suppression by AGS-associated proteins. Indeed, although TREX1 possesses exonuclease activity that can easily target single-stranded LINE-1 cDNA, it preferentially induces ORF1p degradation through proteasome-mediated hydrolysis (95). Similarly, SAMHD1, as the only dNTPase (which degrades dNTP) in human cells, suppresses LINE-1 by reducing the protein levels of ORF2p (70), while ADAR1 simply binds to LINE-1 RNA and inhibits LINE-1 retrotransposition without using its RNA mutagenesis activity (71). Notably, all these mechanisms, along with those of many other endogenous LINE-1 suppressors, target the formation/function of LINE-1 RNP (**Table 1**), suggesting the possibility that LINE-1 RNP might also trigger innate immune activation through another pathway.

**Table 1:** Targets of endogenous LINE-1 suppressors in human cells.

Suppressor	Target(s)	Reference(s)
ADAR1	LINE-1 RNA	(71)
APOBEC3 proteins	ORF1p and ORF2p	(96)
MOV10	LINE-1 RNA	(97, 98)
SAMHD1	ORF1p and ORF2p	(70, 99)
TREX1	ORF1p	(95)
TRIM5α	LINE-1 5’-UTR^a^	(100)
ZAP	LINE-1 RNA	(101, 102)

a LINE-1 RNA synthesis can be suppressed through TRIM5α-mediated inhibition of the promoter activity of the LINE-1 5’-UTR.

Consistent with the above assumption, it was later determined that LINE-1 could increase IFN production in HEK293T and HeLa cells, both of which are defective in the cGAS-mediated DNA-sensing pathway (103). Even in THP-1 cells where the cGAS-STING pathway is active, manipulating the levels of endogenous LINE-1 RNA was found to alter the levels of innate immune activation in a time frame when the levels of endogenous LINE-1 DNA remained unchanged (104). All these phenomena confirmed the existence of at least one cGAS-independent mechanism through which LINE-1 promotes IFN level elevation. Notably, RNA-sensing pathways are effective in HEK293T cells, and knocking down the expression of RNA sensors such as MDA5 or RIG-I indeed compromises LINE-1’s ability to activate the IFN signaling system (**Figure 5**, green pathway). Subsequent research indicated that both MDA5 and RIG-I can recognize and interact with LINE-1 RNA, providing the first direct evidence that LINE-1 can activate sensors of endogenous DNA/RNA-sensing pathways. Interestingly, although RNA sensors “sense” RNA, both LINE-1 proteins (i.e., ORF1p and ORF2p) are also essential for MDA5/RIG-I activation. In fact, it appears that the LINE-1 RNP is the fundamental unit for LINE-1-induced activation of RNA-sensing pathways (104). In summary, LINE-1 has finally been confirmed as an endogenous trigger of innate immune activation.

### LINE-1 Is Regulated by the IFN Signaling System

Being inside mammalian cells for millions of years, LINE-1 has generated a tremendous number of copies that are integrated into genomes from different mammals. For example, there are ~500,000 copies of LINE-1 fragments in a single diploid human cell (105). Fortunately, LINE-1 retrotransposition tends to generate inactive copies, while many other copies have been deactivated through epigenetic mechanisms, such as DNA methylation. As a result, there are currently 80-120 retrotransposition-competent LINE-1 copies in each human cell (106), which are strictly regulated by an intricate network of proteins and microRNAs (10). This arrangement makes perfect sense because the unregulated activity of LINE-1 might over-activate the IFN signaling system and trigger autoimmune disease development. Intriguingly, many members of this network are ISG protein products (**Figure 5**, red pathway). A negative feedback loop is therefore formed: 1) LINE-1 with its formed RNP and synthesized cDNA triggers the activation of the IFN signaling system; 2) innate immune activation leads to an increase in IFN levels and the subsequent elevation of ISG expression; and 3) elevated ISG protein levels result in a more suppressive effect against LINE-1 retrotransposition, thus lowering the latter’s potency in innate immune activation (**Figure 5**). It is thus easy to hypothesize that LINE-1 suppressors might also function as innate immune regulators, which has indeed been proven with not only AGS-associated proteins such as ADAR1 but also other known LINE-1 regulators such as the APOBEC3C and MOV10 proteins (104). On the other hand, this evidence also confirms the possibility that endogenous LINE-1 can be a target for innate immune suppression, leading to new ideas and designs for treatments against autoimmune diseases.

More interestingly, some of these LINE-1 suppressors are also HIV inhibitors. This correspondence is reasonable because, as progenies of integrated ancient retroviruses, LINE-1 should share some similarities with modern retroviruses such as HIV. Indeed, ISG proteins such as MOV10 and ZAP repress HIV and LINE-1 through the same mechanism, by targeting their RNA (97, 98, 101, 102, 107–111). However, as research has extended to more ISG proteins, it is surprising to observe that many of them inhibit HIV and LINE-1 with alternative mechanisms [please refer to (112) for additional details]. For example, dNTPase activity is essential for SAMHD1 to suppress HIV replication (56) but dispensable in SAMHD1-mediated LINE-1 regulation (70, 113); moreover, DNA deamination plays a key role in APOBEC3-induced HIV suppression but is not involved in APOBEC3 inhibiting LINE-1, with APOBEC3A as the only exception (96). There are some possible reasons to explain such phenomena, such as the differences between the replication processes of HIV and LINE-1 and the subcellular localization of restriction factors and HIV/LINE-1 components, while the timing of suppression might be the most important. For instance, APOBEC3 proteins suppress HIV by introducing mutagenesis to the viral genome, while the suppressive effect can be observed only in next-round infection (50). As discussed above, many LINE-1 suppressors also suppress LINE-1-induced innate immune activation. During LINE-1 replication, it forms RNPs, nicks the host genome, and generates cDNA, all of which trigger endogenous sensors and promotes IFN production. With their mutagenesis activity, APOBEC3 proteins in theory can still suppress LINE-1 replication in the next round of retrotransposition but would fail to reduce LINE-1-induced genome damage and IFN production in the current round. With active copies of LINE-1 already being integrated into the human genome (which is different from the case in exogenous HIV infection), an alternative mechanism is therefore necessary. In fact, APOBEC3 proteins interact with ORF1p and/or ORF2p; consequently, compromising the formation/stability/functionality of LINE-1 RNP might be the mechanism by which APOBEC3 proteins suppress LINE-1 replication (96). Notably, such phenomena also indicate that the differences between HIV and LINE-1 are in fact significant enough for host factors to distinguish the two and that it is possible to design an intervention approach specifically targeting HIV without interfering with LINE-1 and its ability to maintain innate immune activation.

## HIV and LINE-1

### HIV Participates in LINE-1 Regulation With Debatable Outcomes

**Figure 6:**
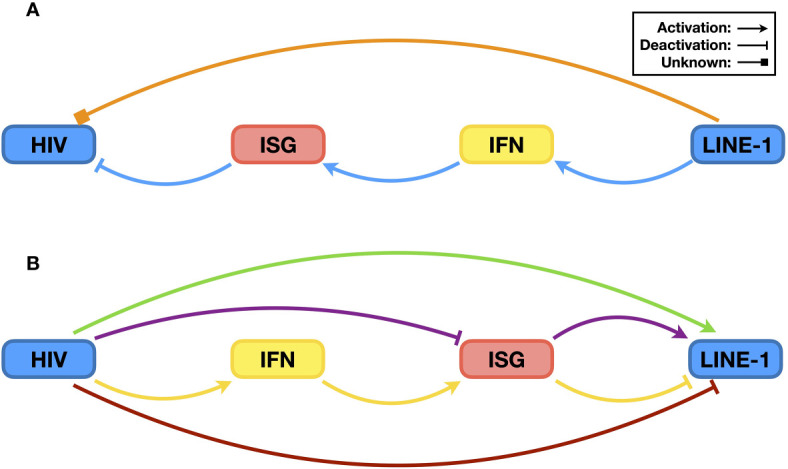
The relationship between HIV and LINE-1. **(A)** Possible impact of LINE-1 on HIV. It has been reported that LINE-1 ORF1p can be packed into HIV virions, but the biological significance is currently unknown. Therefore, it is not clear whether LINE-1 can promote or suppress HIV infectivity directly (orange pathway). However, it has been confirmed that LINE-1 can increase endogenous levels of IFN, leading to the expression of ISGs and subsequent HIV suppression (blue pathway). **(B)** Possible impacts of HIV upon LINE-1 retrotransposition. With a similar ancient retrovirus origin, components of HIV might directly enhance LINE-1 activity (green pathway). In addition, HIV might increase LINE-1 activity by neutralizing ISG proteins that function as both HIV restriction factors and LINE-1 suppressors (purple pathway). On the other hand, HIV infection triggers innate immune activation, and the resulting elevation in ISG protein levels might lead to reduced LINE-1 replication (yellow pathway). Additionally, considering LINE-1 as the trigger of the IFN signaling system, HIV might suppress LINE-1 retrotransposition directly to lower levels of innate immune activation (red pathway).

The differences between HIV and LINE-1 also raise an interesting question: do HIV and LINE-1 act directly on each other? For LINE-1, this might be an easy question to answer because it has a relatively simpler structure and fewer components. In fact, the only known participation of LINE-1 in HIV replication is that LINE-1 ORF1p can be packed into HIV virions (114). However, the associated mechanism or biological significance of this phenomenon remains unknown (**Figure 6A**, orange pathway).

On the other hand, it is clear that HIV does affect LINE-1 retrotransposition, the result of which is, however, debated, and the associated mechanisms are mostly unknown. The very first direct evidence suggesting that HIV infection alters LINE-1 activity was reported in 2013. Jones et al. (115) found that HIV infection promotes LINE-1 retrotransposition in Jurkat cells, a CD4^+^ cell line often used for HIV study *in vitro*. Interestingly, by removing genes from the viral genome, they uncovered that both Vif and Vpr are essential for HIV-induced LINE-1 promotion. This result was understandable because the key function of Vif is to induce the degradation of antiviral proteins from the APOBEC3 family (116), some of which also act as LINE-1 suppressors, as mentioned above (96). Moreover, Vpr is famous for its high potency in arresting the host cell cycle at the G2/M phage (117), which subsequently promotes HIV production (118). LINE-1 may benefit from both activities as a side effect. In addition, Vpr was previously found in the blood of HIV-1 patients (119), and another study reported in 2013 indicated that extracellular recombinant Vpr (rVpr) also functions as a LINE-1 promoter in Huh-7 cells (derived from liver cancer) transgenic mice harboring human LINE-1 (120), and RAW264.7 (macrophage-like) cells (121). Sadly, however, no Vif or Vpr mutant was tested, leading to the obscurity of associated mechanisms.

While people have started to believe that the expression of HIV components benefits LINE-1 retrotransposition, different opinions have been presented regarding the Vpr protein. In 2018, Kawano et al. (114) published a study suggesting that Vpr actually suppresses LINE-1 replication. With the change to HEK293T cells that are usually used in LINE-1 retrotransposition assays, they found that transfection (instead of infection as previously done) of several HIV proviral plasmids resulted in similar effective suppression of LINE-1 activity. Moreover, expressing Vpr inside eukaryotic HEK293T cells (rather than extracellular treatment of Vpr purified from prokaryotic *E. coli* cells) led to potent inhibition of LINE-1 replication. Most importantly, they proposed a mechanism by which Vpr interacts with LINE-1 ORF2p and suppresses its reverse transcriptase activity. These different results, although likely due to the different designs of experiments, obscure the idea that HIV promotes LINE-1 replication.

### The Relationship Between HIV and LINE-1 Is Complicated by the IFN Signaling System

Whether HIV suppresses or enhances LINE-1 activity is further complicated by the involvement of the IFN signaling system, and it seems that acting on LINE-1 itself might cause a dilemma for HIV (**Figure 6**). It is now clear that by inducing IFN production, the components and activities of LINE-1 contribute to host antiviral defense (**Figure 6A**, blue pathway). Indeed, manipulating LINE-1 RNA levels in THP-1 cells affects HIV infectivity (104). Similarly, HIV-mediated activation of the IFN signaling system might also lead to LINE-1 suppression due to elevated levels of ISG proteins (**Figure 6B**, yellow pathway). However, to achieve high infectivity, HIV needs to reduce the levels of ISG expression. Accordingly, it is reasonable that HIV might suppress LINE-1 activity to reduce the activation level of the IFN signaling system (**Figure 6B**, red pathway). On the other hand, although genetically distinct from HIV, LINE-1 is still considered a residue of ancient retroviruses (10). Therefore, it is also possible that LINE-1 might benefit from the presence of proteins of modern retroviruses, such as HIV (**Figure 6B**, green pathway).

In addition, as mentioned before, many restriction factors suppressing HIV also function as LINE-1 inhibitors [for additional information, please refer to (10)]. In theory, viral proteins that compromise these factors should enhance LINE-1 activity (**Figure 6B**, purple pathway). HIV Vif promoting LINE-1 retrotransposition through the depletion of APOBEC3 proteins is a perfect example (115). Another example is that Vpx, a viral protein specifically expressed from HIV-2 and some SIVs, increases LINE-1 replication efficiency by reducing the protein levels of the restriction factor SAMHD1 (70). Surprisingly, however, a similar scenario has not been observed with other HIV proteins. For instance, BST2 is an antiviral protein that restricts enveloped viruses on producer cells and prevents viral release (53). To counteract such an antiviral mechanism, HIV utilizes its Vpu protein to remove BST2 from the cell surface and induce its degradation (54, 55). On the other hand, BST2 is also capable of LINE-1 inhibition (101). Thus, Vpu might enhance LINE-1 replication by reducing BST2 levels. Study results, however, have indicated that although comprising BST2 could result in a rescue of LINE-1 retrotransposition, Vpu by itself in fact functions as a potent LINE-1 suppressor (101); however, the latter part does correlate with the previous observation that Vpu suppresses IFN production in infected cells (122). In other words, Vpu at proper levels might promote LINE-1 activity through BST2 reduction, but if expressed at higher levels, Vpu will suppress LINE-1 retrotransposition. The situation is further complicated by the fact that the expression of Vpu can be affected in multiple ways, such as the efficiency of viral promoters, codon usage of the *vpu* gene, and host regulation of gene expression, along with the fact that Vpu proteins from different HIV subtypes or strains might have different potency against LINE-1 activity due to variations in protein sequences. Considering the many proteins expressed by the virus, cases such as that for Vpu might explain why different effects have been observed for HIV regulating LINE-1, which also make it unpredictable how HIV affects LINE-1 in general. Taken together, these data suggest that an interaction between HIV and LINE-1 does exist, the details of which, however, require more extensive study.

## Perspectives

Since HIV is a type of exogenous virus and LINE-1 is derived from ancient viruses, it is easy to understand that both can activate the IFN signaling system. Because both generate RNA and require reverse transcription for their replication, it is also understandable that HIV and LINE-1 trigger the activation of endogenous DNA and RNA sensors. On the other hand, given that HIV is the causative reagent of AIDS and that un-regulated LINE-1 is associated with autoimmune diseases, it also makes perfect sense that innate immune activation results in the suppression of both. However, the relationship between HIV and LINE-1 is complex, especially when the IFN signaling system is also involved. For example, considering the facts that both HIV and LINE-1 are derived from ancient viruses (though possibly from different types) and that both involve reverse transcription, it is not bizarre to speculate that one would enhance the replication of the other. In addition, to promote replication, HIV suppresses the activation of the IFN signaling system and induces the depletion of restriction factors, which should also benefit LINE-1 retrotransposition since many restriction factors also function as LINE-1 suppressors. On the other hand, LINE-1 and its activity trigger innate immune activation that would suppress HIV infection; therefore, it is reasonable for HIV to inhibit LINE-1 replication. Moreover, HIV infection by itself induces the activation of the IFN signaling system, which can also lead to LINE-1 suppression. All these possibilities might be reasons for the inconsistency among published studies on how HIV regulates LINE-1. Apparently, the details on the relationship between HIV and LINE-1 need to be investigated completely and carefully.

It is, however, surprising to find that despite the many similarities between HIV and LINE-1, it seems possible to separate one from the other. The most convincing data are that restriction factors use different mechanisms to suppress HIV and LINE-1. Additionally, viral proteins such as Vpr and Vpu that benefit HIV replication can inhibit LINE-1 retrotransposition. Furthermore, a recent study indicated that even for the reverse transcription aspect that is shared by both HIV and LINE-1, specific inhibitors can be synthesized (123). Such phenomena provide opportunities for developing novel drugs against not only HIV infection but also autoimmune diseases. Since the regulation of LINE-1 and HIV can be separated, if one can recreate the situation where LINE-1 is overactivated without the simultaneous promotion of HIV infection, then LINE-1-triggered innate immune activation should generate host antiviral defense that would suppress HIV replication. For instance, the formation of LINE-1 RNP is essential for the activation of RNA sensors like RIG-I and MDA5 (104). In other words, the motif on LINE-1 RNA that activates RNA sensing pathway is most likely hidden in the secondary structure of LINE-1 RNA and can only be exposed once the RNP is formed. Accordingly, if one can determine the sequence of this motif and introduce it into human cells, or if one can design a small molecule that can enter human cells and expose this motif even without the interaction of LINE-1 proteins, the levels of innate immune activation would be increased. Consequently, the resulted elevation of IFNs and subsequent expression of ISG proteins should almost definitely enhance the antiviral potency of the patient and form a strengthened barrier against not only HIV replication but also possibly the opportunistic infections that lead to AIDS. This might be an interesting strategy against HIV infection and AIDS development because it is not a treatment directly targeting HIV components; HIV cannot evade *via* mutagenesis, as it does against anti-HIV drugs, such as NRTIs and NNRTIs.

On the other hand, it has already been proven that many LINE-1 suppressors also function as innate immune regulators through LINE-1 inhibition (104). Notably, components of HIV (such as Vpu and possibly Vpr) have been confirmed to inhibit LINE-1 retrotransposition (101, 114). It is therefore possible that these viral components might also reduce the activation levels of the IFN signaling system. If true, these viral components and associated mechanism(s) for LINE-1 suppression might provide molecular bases for developing drugs to repress autoimmunity. Currently, however, it is difficult to propose novel Vpr- or Vpu-based therapies on autoimmune diseases because critical information is still missing. It has been revealed that, Vpr suppresses LINE-1 by interacting with ORF2p and reducing the latter’s reverse transcriptase activity (114); but whether Vpr represses the IFN signaling system through LINE-1 inhibition remains unknown. Similarly, Vpu suppresses LINE-1 (101) and reduces IFN production (122); however, it is not clear whether the two phenomena are related, while associated mechanisms are also not understood. Future research efforts on the interplay among HIV, LINE-1, and the IFN signaling system are therefore worthwhile, as new information is waiting to be revealed.

## Author Contributions

KZ chose the topic. All authors discussed and wrote the manuscript, with XZ and YZ contributing equally. All authors contributed to the article and approved the submitted version.

## Funding

Our work was funded in part by grants from the National Natural Science Foundation of China (81601363 and 82071853), Fundamental Research Funds for the Central Universities (2017TD-08), China Postdoctoral Science Foundation (2020M670843), National Natural Science Foundation of Jilin Province (JLSCZD2019-008), Key Laboratory of Molecular Virology, Jilin Province (20102209), Norman Bethune Health Science Center of Jilin University (2018B18), and First Hospital of Jilin University (2020-CXQ-02).

## Conflict of Interest

The authors declare that the research was conducted in the absence of any commercial or financial relationships that could be construed as a potential conflict of interest.

## Publisher’s Note

All claims expressed in this article are solely those of the authors and do not necessarily represent those of their affiliated organizations, or those of the publisher, the editors and the reviewers. Any product that may be evaluated in this article, or claim that may be made by its manufacturer, is not guaranteed or endorsed by the publisher.

## References

[1] Walter MR. The Role of Structure in the Biology of Interferon Signaling. Front Immunol (2020) 11:606489. 10.3389/fimmu.2020.606489 33281831 PMC7689341

[2] Liu J Qian C Cao X. Post-Translational Modification Control of Innate Immunity. Immunity (2016) 45:15–30. 10.1016/j.immuni.2016.06.020 27438764

[3] Lazear HM Schoggins JW Diamond MS. Shared and Distinct Functions of Type I and Type III Interferons. Immunity (2019) 50:907–23. 10.1016/j.immuni.2019.03.025 PMC6839410 30995506

[4] Schoggins JW Wilson SJ Panis M Murphy MY Jones CT Bieniasz P. A Diverse Range of Gene Products Are Effectors of the Type I Interferon Antiviral Response. Nature (2011) 472:481–5. 10.1038/nature09907 PMC3409588 21478870

[5] Schneider WM Chevillotte MD Rice CM. Interferon-Stimulated Genes: A Complex Web of Host Defenses. Annu Rev Immunol (2014) 32:513–45. 10.1146/annurev-immunol-032713-120231 PMC4313732 24555472

[6] Schoggins JW. Interferon-Stimulated Genes: What Do They All Do? Annu Rev Virol (2019) 6:567–84. 10.1146/annurev-virology-092818-015756 31283436

[7] Crow MK Olferiev M Kirou KA. Type I Interferons in Autoimmune Disease. Annu Rev Pathol (2019) 14:369–93. 10.1146/annurev-pathol-020117-043952 30332560

[8] Crow MK. Long Interspersed Nuclear Elements (LINE-1): Potential Triggers of Systemic Autoimmune Disease. Autoimmunity (2010) 43:7–16. 10.3109/08916930903374865 19961365

[9] Volkman HE Stetson DB. The Enemy Within: Endogenous Retroelements and Autoimmune Disease. Nat Immunol (2014) 15:415–22. 10.1038/ni.2872 PMC4131434 24747712

[10] Goodier JL. Restricting Retrotransposons: A Review. Mob DNA (2016) 7:16. 10.1186/s13100-016-0070-z 27525044 PMC4982230

[11] Ostertag EM Kazazian HH Jr. Biology of Mammalian L1 Retrotransposons. Annu Rev Genet (2001) 35:501–38. 10.1146/annurev.genet.35.102401.091032 11700292

[12] Barre-Sinoussi F Chermann JC Rey F Nugeyre MT Chamaret S Gruest J. Isolation of a T-Lymphotropic Retrovirus From a Patient at Risk for Acquired Immune Deficiency Syndrome (AIDS). Science (1983) 220:868–71. 10.1126/science.6189183 6189183

[13] Gallo RC Sarin PS Gelmann EP Robert-Guroff M Richardson E Kalyanaraman VS. Isolation of Human T-Cell Leukemia Virus in Acquired Immune Deficiency Syndrome (AIDS). Science (1983) 220:865–7. 10.1126/science.6601823 6601823

[14] Engelman A Cherepanov P. The Structural Biology of HIV-1: Mechanistic and Therapeutic Insights. Nat Rev Microbiol (2012) 10:279–90. 10.1038/nrmicro2747 PMC3588166 22421880

[15] Rosen CA Sodroski JG Haseltine WA. Location of Cis-Acting Regulatory Sequences in the Human T-Cell Leukemia Virus Type I Long Terminal Repeat. Proc Natl Acad Sci USA (1985) 82:6502–6. 10.1073/pnas.82.19.6502 PMC390745 2995968

[16] Dayton AI Sodroski JG Rosen CA Goh WC Haseltine WA. The Trans-Activator Gene of the Human T Cell Lymphotropic Virus Type III Is Required for Replication. Cell (1986) 44:941–7. 10.1016/0092-8674(86)90017-6 2420471

[17] Fisher AG Feinberg MB Josephs SF Harper ME Marselle LM Reyes G. The Trans-Activator Gene of HTLV-III Is Essential for Virus Replication. Nature (1986) 320:367–71. 10.1038/320367a0 3007995

[18] Sodroski J Goh WC Rosen C Dayton A Terwilliger E Haseltine W. A Second Post-Transcriptional Trans-Activator Gene Required for HTLV-III Replication. Nature (1986) 321:412–7. 10.1038/321412a0 3012355

[19] Fang X Wang J O’Carroll IP Mitchell M Zuo X Wang Y. An Unusual Topological Structure of the HIV-1 Rev Response Element. Cell (2013) 155:594–605. 10.1016/j.cell.2013.10.008 24243017 PMC3918456

[20] Fisher AG Ratner L Mitsuya H Marselle LM Harper ME Broder S. Infectious Mutants of HTLV-III With Changes in the 3’ Region and Markedly Reduced Cytopathic Effects. Science (1986) 233:655–9. 10.1126/science.3014663 3014663

[21] Dedera D Hu W Vander Heyden N Ratner L. Viral Protein R of Human Immunodeficiency Virus Types 1 and 2 Is Dispensable for Replication and Cytopathogenicity in Lymphoid Cells. J Virol (1989) 63:3205–8. 10.1128/JVI.63.7.3205-3208.1989 PMC250884 2524599

[22] Sakai H Shibata R Sakuragi J Sakuragi S Kawamura M Adachi A. Cell-Dependent Requirement of Human Immunodeficiency Virus Type 1 Vif Protein for Maturation of Virus Particles. J Virol (1993) 67:1663–6. 10.1128/JVI.67.3.1663-1666.1993 PMC237539 8437236

[23] Sheehy AM Gaddis NC Choi JD Malim MH. Isolation of a Human Gene That Inhibits HIV-1 Infection and Is Suppressed by the Viral Vif Protein. Nature (2002) 418:646–50. 10.1038/nature00939 12167863

[24] Mariani R Chen D Schrofelbauer B Navarro F Konig R Bollman B. Species-Specific Exclusion of APOBEC3G From HIV-1 Virions by Vif. Cell (2003) 114:21–31. 10.1016/s0092-8674(03)00515-4 12859895

[25] Marin M Rose KM Kozak SL Kabat D. HIV-1 Vif Protein Binds the Editing Enzyme APOBEC3G and Induces Its Degradation. Nat Med (2003) 9:1398–403. 10.1038/nm946 14528301

[26] Sheehy AM Gaddis NC Malim MH. The Antiretroviral Enzyme APOBEC3G Is Degraded by the Proteasome in Response to HIV-1 Vif. Nat Med (2003) 9:1404–7. 10.1038/nm945 14528300

[27] Yu X Yu Y Liu B Luo K Kong W Mao P. Induction of APOBEC3G Ubiquitination and Degradation by an HIV-1 Vif-Cul5-SCF Complex. Science (2003) 302:1056–60. 10.1126/science.1089591 14564014

[28] Neil SJ Eastman SW Jouvenet N Bieniasz PD. HIV-1 Vpu Promotes Release and Prevents Endocytosis of Nascent Retrovirus Particles From the Plasma Membrane. PLoS Pathog (2006) 2:e39. 10.1371/journal.ppat.0020039 16699598 PMC1458960

[29] Hrecka K Hao C Gierszewska M Swanson SK Kesik-Brodacka M Srivastava S. Vpx Relieves Inhibition of HIV-1 Infection of Macrophages Mediated by the SAMHD1 Protein. Nature (2011) 474:658–61. 10.1038/nature10195 PMC3179858 21720370

[30] Laguette N Sobhian B Casartelli N Ringeard M Chable-Bessia C Segeral E. SAMHD1 Is the Dendritic- and Myeloid-Cell-Specific HIV-1 Restriction Factor Counteracted by Vpx. Nature (2011) 474:654–7. 10.1038/nature10117 PMC3595993 21613998

[31] Rosa A Chande A Ziglio S De Sanctis V Bertorelli R Goh SL. HIV-1 Nef Promotes Infection by Excluding SERINC5 From Virion Incorporation. Nature (2015) 526:212–7. 10.1038/nature15399 PMC4861059 26416734

[32] Usami Y Wu Y Gottlinger HG. SERINC3 and SERINC5 Restrict HIV-1 Infectivity and Are Counteracted by Nef. Nature (2015) 526:218–23. 10.1038/nature15400 PMC4600458 26416733

[33] Morris KL Buffalo CZ Sturzel CM Heusinger E Kirchhoff F Ren X. HIV-1 Nefs Are Cargo-Sensitive AP-1 Trimerization Switches in Tetherin Downregulation. Cell (2018) 174:659–71.e14. 10.1016/j.cell.2018.07.004 30053425 PMC6091687

[34] Harris RS Liddament MT. Retroviral Restriction by APOBEC Proteins. Nat Rev Immunol (2004) 4:868–77. 10.1038/nri1489 15516966

[35] Nakayama EE Shioda T. TRIM5alpha and Species Tropism of HIV/SIV. Front Microbiol (2012) 3:13. 10.3389/fmicb.2012.00013 22291694 PMC3264904

[36] Goujon C Moncorge O Bauby H Doyle T Ward CC Schaller T. Human MX2 Is an Interferon-Induced Post-Entry Inhibitor of HIV-1 Infection. Nature (2013) 502:559–62. 10.1038/nature12542 PMC3808269 24048477

[37] Kane M Yadav SS Bitzegeio J Kutluay SB Zang T Wilson SJ. MX2 Is an Interferon-Induced Inhibitor of HIV-1 Infection. Nature (2013) 502:563–6. 10.1038/nature12653 PMC3912734 24121441

[38] Liu Z Pan Q Ding S Qian J Xu F Zhou J. The Interferon-Inducible Mxb Protein Inhibits HIV-1 Infection. Cell Host Microbe (2013) 14:398–410. 10.1016/j.chom.2013.08.015 24055605

[39] Mangeat B Gers-Huber G Lehmann M Zufferey M Luban J Piguet V. HIV-1 Vpu Neutralizes the Antiviral Factor Tetherin/BST-2 by Binding It and Directing Its Beta-TrCP2-Dependent Degradation. PLoS Pathog (2009) 5:e1000574. 10.1371/journal.ppat.1000574 19730691 PMC2729927

[40] Rollason R Dunstan K Billcliff PG Bishop P Gleeson P Wise H. Expression of HIV-1 Vpu Leads to Loss of the Viral Restriction Factor Cd317/Tetherin From Lipid Rafts and Its Enhanced Lysosomal Degradation. PLoS One (2013) 8:e75680. 10.1371/journal.pone.0075680 24086611 PMC3782430

[41] Su J Rui Y Lou M Yin L Xiong H Zhou Z. HIV-2/Siv Vpx Targets a Novel Functional Domain of Sting to Selectively Inhibit cGAS-Sting-Mediated Nf-Kappab Signalling. Nat Microbiol (2019) 4:2552–64. 10.1038/s41564-019-0585-4 31659299

[42] Kato H Takeuchi O Sato S Yoneyama M Yamamoto M Matsui K. Differential Roles of MDA5 and RIG-I Helicases in the Recognition of RNA Viruses. Nature (2006) 441:101–5. 10.1038/nature04734 16625202

[43] Solis M Nakhaei P Jalalirad M Lacoste J Douville R Arguello M. RIG-I-Mediated Antiviral Signaling Is Inhibited in HIV-1 Infection by a Protease-Mediated Sequestration of RIG-I. J Virol (2011) 85:1224–36. 10.1128/JVI.01635-10 PMC3020501 21084468

[44] Osei Kuffour E Konig R Haussinger D Schulz WA Munk C. Isg15 Deficiency Enhances HIV-1 Infection by Accumulating Misfolded P53. mBio (2019) 10:1–15. 10.1128/mBio.01342-19 PMC6712392 31455647

[45] Yan N Regalado-Magdos AD Stiggelbout B Lee-Kirsch MA Lieberman J. The Cytosolic Exonuclease TREX1 Inhibits the Innate Immune Response to Human Immunodeficiency Virus Type 1. Nat Immunol (2010) 11:1005–13. 10.1038/ni.1941 PMC2958248 20871604

[46] Mazur DJ Perrino FW. Identification and Expression of the TREX1 and TREX2 cDNA Sequences Encoding Mammalian 3’–>5’ Exonucleases. J Biol Chem (1999) 274:19655–60. 10.1074/jbc.274.28.19655 10391904

[47] Gao D Wu J Wu YT Du F Aroh C Yan N. Cyclic Gmp-Amp Synthase Is an Innate Immune Sensor of HIV and Other Retroviruses. Science (2013) 341:903–6. 10.1126/science.1240933 PMC3860819 23929945

[48] Sun L Wu J Du F Chen X Chen ZJ. Cyclic Gmp-Amp Synthase Is a Cytosolic DNA Sensor That Activates the Type I Interferon Pathway. Science (2013) 339:786–91. 10.1126/science.1232458 PMC3863629 23258413

[49] Wu J Sun L Chen X Du F Shi H Chen C. Cyclic Gmp-Amp Is an Endogenous Second Messenger in Innate Immune Signaling by Cytosolic DNA. Science (2013) 339:826–30. 10.1126/science.1229963 PMC3855410 23258412

[50] Harris RS Bishop KN Sheehy AM Craig HM Petersen-Mahrt SK Watt IN. DNA Deamination Mediates Innate Immunity to Retroviral Infection. Cell (2003) 113:803–9. 10.1016/s0092-8674(03)00423-9 12809610

[51] Ward AE Kiessling V Pornillos O White JM Ganser-Pornillos BK Tamm LK. HIV-Cell Membrane Fusion Intermediates Are Restricted by Serincs as Revealed by Cryo-Electron and TIRF Microscopy. J Biol Chem (2020) 295:15183–95. 10.1074/jbc.RA120.014466 PMC7650252 32788212

[52] Chen YC Sood C Marin M Aaron J Gratton E Salaita K. Super-Resolution Fluorescence Imaging Reveals That Serine Incorporator Protein 5 Inhibits Human Immunodeficiency Virus Fusion by Disrupting Envelope Glycoprotein Clusters. ACS Nano (2020) 14:10929–43. 10.1021/acsnano.0c02699 PMC8274448 32441921

[53] Perez-Caballero D Zang T Ebrahimi A McNatt MW Gregory DA Johnson MC. Tetherin Inhibits HIV-1 Release by Directly Tethering Virions to Cells. Cell (2009) 139:499–511. 10.1016/j.cell.2009.08.039 19879838 PMC2844890

[54] Neil SJ Zang T Bieniasz PD. Tetherin Inhibits Retrovirus Release and Is Antagonized by HIV-1 Vpu. Nature (2008) 451:425–30. 10.1038/nature06553 18200009

[55] Van Damme N Goff D Katsura C Jorgenson RL Mitchell R Johnson MC. The Interferon-Induced Protein Bst-2 Restricts HIV-1 Release and Is Downregulated From the Cell Surface by the Viral Vpu Protein. Cell Host Microbe (2008) 3:245–52. 10.1016/j.chom.2008.03.001 PMC2474773 18342597

[56] Lahouassa H Daddacha W Hofmann H Ayinde D Logue EC Dragin L. SAMHD1 Restricts the Replication of Human Immunodeficiency Virus Type 1 by Depleting the Intracellular Pool of Deoxynucleoside Triphosphates. Nat Immunol (2012) 13:223–8. 10.1038/ni.2236 PMC3771401 22327569

[57] St Gelais C de Silva S Amie SM Coleman CM Hoy H Hollenbaugh JA. SAMHD1 Restricts HIV-1 Infection in Dendritic Cells (Dcs) by Dntp Depletion, But Its Expression in DCS and Primary CD4+ T-Lymphocytes Cannot Be Upregulated by Interferons. Retrovirology (2012) 9:105. 10.1186/1742-4690-9-105 23231760 PMC3527137

[58] Jauregui P Logue EC Schultz ML Fung S Landau NR. Degradation of SAMHD1 by Vpx Is Independent of Uncoating. J Virol (2015) 89:5701–13. 10.1128/JVI.03575-14 PMC4442547 25762741

[59] Hu Y Desimmie BA Nguyen HC Ziegler SJ Cheng TC Chen J. Structural Basis of Antagonism of Human Apobec3f by HIV-1 Vif. Nat Struct Mol Biol (2019) 26:1176–83. 10.1038/s41594-019-0343-6 PMC6899190 31792451

[60] Salamango DJ Ikeda T Moghadasi SA Wang J McCann JL Serebrenik AA. HIV-1 Vif Triggers Cell Cycle Arrest by Degrading Cellular PPP2R5 Phospho-Regulators. Cell Rep (2019) 29:1057–65.e4. 10.1016/j.celrep.2019.09.057 31665623 PMC6903395

[61] Aicardi J Goutieres F. A Progressive Familial Encephalopathy in Infancy With Calcifications of the Basal Ganglia and Chronic Cerebrospinal Fluid Lymphocytosis. Ann Neurol (1984) 15:49–54. 10.1002/ana.410150109 6712192

[62] Crow YJ Manel N. Aicardi-Goutieres Syndrome and the Type I Interferonopathies. Nat Rev Immunol (2015) 15:429–40. 10.1038/nri3850 26052098

[63] Crow YJ Hayward BE Parmar R Robins P Leitch A Ali M. Mutations in the Gene Encoding the 3’-5’ DNA Exonuclease TREX1 Cause Aicardi-Goutieres Syndrome at the Ags1 Locus. Nat Genet (2006) 38:917–20. 10.1038/ng1845 16845398

[64] Yang YG Lindahl T Barnes DE. TREX1 Exonuclease Degrades ssDNA to Prevent Chronic Checkpoint Activation and Autoimmune Disease. Cell (2007) 131:873–86. 10.1016/j.cell.2007.10.017 18045533

[65] Stetson DB Ko JS Heidmann T Medzhitov R. TREX1 Prevents Cell-Intrinsic Initiation of Autoimmunity. Cell (2008) 134:587–98. 10.1016/j.cell.2008.06.032 PMC2626626 18724932

[66] Crow YJ Leitch A Hayward BE Garner A Parmar R Griffith E. Mutations in Genes Encoding Ribonuclease H2 Subunits Cause Aicardi-Goutieres Syndrome and Mimic Congenital Viral Brain Infection. Nat Genet (2006) 38:910–6. 10.1038/ng1842 16845400

[67] Rice GI Bond J Asipu A Brunette RL Manfield IW Carr IM. Mutations Involved in Aicardi-Goutieres Syndrome Implicate SAMHD1 as Regulator of the Innate Immune Response. Nat Genet (2009) 41:829–32. 10.1038/ng.373 PMC4154505 19525956

[68] Rice GI Kasher PR Forte GM Mannion NM Greenwood SM Szynkiewicz M. Mutations in Adar1 Cause Aicardi-Goutieres Syndrome Associated With a Type I Interferon Signature. Nat Genet (2012) 44:1243–8. 10.1038/ng.2414 PMC4154508 23001123

[69] Rice GI Del Toro Duany Y Jenkinson EM Forte GM Anderson BH Ariaudo G. Gain-of-Function Mutations in IFIH1 Cause a Spectrum of Human Disease Phenotypes Associated With Upregulated Type I Interferon Signaling. Nat Genet (2014) 46:503–9. 10.1038/ng.2933 PMC4004585 24686847

[70] Zhao K Du J Han X Goodier JL Li P Zhou X. Modulation of LINE-1 and Alu/SVA Retrotransposition by Aicardi-Goutieres Syndrome-Related SAMHD1. Cell Rep (2013) 4:1108–15. 10.1016/j.celrep.2013.08.019 PMC3988314 24035396

[71] Orecchini E Doria M Antonioni A Galardi S Ciafre SA Frassinelli L. Adar1 Restricts LINE-1 Retrotransposition. Nucleic Acids Res (2017) 45:155–68. 10.1093/nar/gkw834 PMC5224506 27658966

[72] Benitez-Guijarro M Lopez-Ruiz C Tarnauskaite Z Murina O Mian Mohammad M Williams TC. RNAse H2, Mutated in Aicardi-Goutieres Syndrome, Promotes LINE-1 Retrotransposition. EMBO J (2018) 37:1–22. 10.15252/embj.201798506 29959219 PMC6068448

[73] Choi J Hwang SY Ahn K. Interplay Between RNASEH2 and MOV10 Controls LINE-1 Retrotransposition. Nucleic Acids Res (2018) 46:1912–26. 10.1093/nar/gkx1312 PMC5829647 29315404

[74] Adams JW Kaufman RE Kretschmer PJ Harrison M Nienhuis AW. A Family of Long Reiterated DNA Sequences, One Copy of Which Is Next to the Human Beta Globin Gene. Nucleic Acids Res (1980) 8:6113–28. 10.1093/nar/8.24.6113 PMC328076 6258162

[75] Grimaldi G Skowronski J Singer MF. Defining the Beginning and End of Kpni Family Segments. EMBO J (1984) 3:1753–9. 10.1002/j.1460-2075.1984.tb02042.x PMC557592 6090124

[76] Goodier JL Cheung LE Kazazian HH Jr. Mapping the LINE1 ORF1 Protein Interactome Reveals Associated Inhibitors of Human Retrotransposition. Nucleic Acids Res (2013) 41:7401–19. 10.1093/nar/gkt512 PMC3753637 23749060

[77] Taylor MS LaCava J Mita P Molloy KR Huang CR Li D. Affinity Proteomics Reveals Human Host Factors Implicated in Discrete Stages of LINE-1 Retrotransposition. Cell (2013) 155:1034–48. 10.1016/j.cell.2013.10.021 PMC3904357 24267889

[78] Mathias SL Scott AF Kazazian HH Jr. Boeke JD Gabriel A. Reverse Transcriptase Encoded by a Human Transposable Element. Science (1991) 254:1808–10. 10.1126/science.1722352 1722352

[79] Feng Q Moran JV Kazazian HH Jr. Boeke JD. Human L1 Retrotransposon Encodes a Conserved Endonuclease Required for Retrotransposition. Cell (1996) 87:905–16. 10.1016/s0092-8674(00)81997-2 8945517

[80] Denli AM Narvaiza I Kerman BE Pena M Benner C Marchetto MC. Primate-Specific ORF0 Contributes to Retrotransposon-Mediated Diversity. Cell (2015) 163:583–93. 10.1016/j.cell.2015.09.025 26496605

[81] Ablasser A Hemmerling I Schmid-Burgk JL Behrendt R Roers A Hornung V. TREX1 Deficiency Triggers Cell-Autonomous Immunity in a cGAS-Dependent Manner. J Immunol (2014) 192:5993–7. 10.4049/jimmunol.1400737 24813208

[82] Gray EE Treuting PM Woodward JJ Stetson DB. Cutting Edge: cGAS Is Required for Lethal Autoimmune Disease in the TREX1-Deficient Mouse Model of Aicardi-Goutieres Syndrome. J Immunol (2015) 195:1939–43. 10.4049/jimmunol.1500969 PMC4546858 26223655

[83] Belgnaoui SM Gosden RG Semmes OJ Haoudi A. Human LINE-1 Retrotransposon Induces DNA Damage and Apoptosis in Cancer Cells. Cancer Cell Int (2006) 6:13. 10.1186/1475-2867-6-13 16670018 PMC1464142

[84] Gasior SL Wakeman TP Xu B Deininger PL. The Human LINE-1 Retrotransposon Creates DNA Double-Strand Breaks. J Mol Biol (2006) 357:1383–93. 10.1016/j.jmb.2006.01.089 PMC4136747 16490214

[85] Coquel F Silva MJ Techer H Zadorozhny K Sharma S Nieminuszczy J. SAMHD1 Acts at Stalled Replication Forks to Prevent Interferon Induction. Nature (2018) 557:57–61. 10.1038/s41586-018-0050-1 29670289

[86] Mavragani CP Sagalovskiy I Guo Q Nezos A Kapsogeorgou EK Lu P. Expression of Long Interspersed Nuclear Element 1 Retroelements and Induction of Type I Interferon in Patients With Systemic Autoimmune Disease. Arthritis Rheumatol (2016) 68:2686–96. 10.1002/art.39795 PMC5083133 27338297

[87] Perl A. Editorial: Lineing Up to Boost Interferon Production: Activation of Endogenous Retroviral DNA in Autoimmunity. Arthritis Rheumatol (2016) 68:2568–70. 10.1002/art.39794 PMC5083194 27338170

[88] De Cecco M Ito T Petrashen AP Elias AE Skvir NJ Criscione SW. L1 Drives IFN in Senescent Cells and Promotes Age-Associated Inflammation. Nature (2019) 566:73–8. 10.1038/s41586-018-0784-9 PMC6519963 30728521

[89] Merluzzi VJ Hargrave KD Labadia M Grozinger K Skoog M Wu JC. Inhibition of HIV-1 Replication by a Nonnucleoside Reverse Transcriptase Inhibitor. Science (1990) 250:1411–3. 10.1126/science.1701568 1701568

[90] Gulick RM Mellors JW Havlir D Eron JJ Gonzalez C McMahon D. Treatment With Indinavir, Zidovudine, and Lamivudine in Adults With Human Immunodeficiency Virus Infection and Prior Antiretroviral Therapy. N Engl J Med (1997) 337:734–9. 10.1056/NEJM199709113371102 9287228

[91] Hammer SM Squires KE Hughes MD Grimes JM Demeter LM Currier JS. A Controlled Trial of Two Nucleoside Analogues Plus Indinavir in Persons With Human Immunodeficiency Virus Infection and CD4 Cell Counts of 200 Per Cubic Millimeter or Less. Aids Clinical Trials Group 320 Study Team. N Engl J Med (1997) 337:725–33. 10.1056/NEJM199709113371101 9287227

[92] Jones RB Garrison KE Wong JC Duan EH Nixon DF Ostrowski MA. Nucleoside Analogue Reverse Transcriptase Inhibitors Differentially Inhibit Human LINE-1 Retrotransposition. PLoS One (2008) 3:e1547. 10.1371/journal.pone.0001547 18253495 PMC2212136

[93] Beck-Engeser GB Eilat D Wabl M. An Autoimmune Disease Prevented by Anti-Retroviral Drugs. Retrovirology (2011) 8:91. 10.1186/1742-4690-8-91 22067273 PMC3264515

[94] Fowler BJ Gelfand BD Kim Y Kerur N Tarallo V Hirano Y. Nucleoside Reverse Transcriptase Inhibitors Possess Intrinsic Anti-Inflammatory Activity. Science (2014) 346:1000–3. 10.1126/science.1261754 PMC4274127 25414314

[95] Li P Du J Goodier JL Hou J Kang J Kazazian HH Jr. Aicardi-Goutieres Syndrome Protein TREX1 Suppresses L1 and Maintains Genome Integrity Through Exonuclease-Independent ORF1p Depletion. Nucleic Acids Res (2017) 45:4619–31. 10.1093/nar/gkx178 PMC5416883 28334850

[96] Renner TM Belanger K Goodwin LR Campbell M Langlois MA. Characterization of Molecular Attributes That Influence LINE-1 Restriction by All Seven Human Apobec3 Proteins. Virology (2018) 520:127–36. 10.1016/j.virol.2018.05.015 29860216

[97] Goodier JL Cheung LE Kazazian HH Jr. MOV10 RNA Helicase Is a Potent Inhibitor of Retrotransposition in Cells. PLoS Genet (2012) 8:e1002941. 10.1371/journal.pgen.1002941 23093941 PMC3475670

[98] Li X Zhang J Jia R Cheng V Xu X Qiao W. The MOV10 Helicase Inhibits LINE-1 Mobility. J Biol Chem (2013) 288:21148–60. 10.1074/jbc.M113.465856 PMC3774381 23754279

[99] Hu S Li J Xu F Mei S Le Duff Y Yin L. SAMHD1 Inhibits LINE-1 Retrotransposition by Promoting Stress Granule Formation. PLoS Genet (2015) 11:e1005367. 10.1371/journal.pgen.1005367 26134849 PMC4489885

[100] Volkmann B Wittmann S Lagisquet J Deutschmann J Eissmann K Ross JJ. Human TRIM5alpha Senses and Restricts LINE-1 Elements. Proc Natl Acad Sci USA (2020) 117:17965–76. 10.1073/pnas.1922366117 PMC7395565 32651277

[101] Goodier JL Pereira GC Cheung LE Rose RJ Kazazian HH Jr. The Broad-Spectrum Antiviral Protein ZAP Restricts Human Retrotransposition. PLoS Genet (2015) 11:e1005252. 10.1371/journal.pgen.1005252 26001115 PMC4441479

[102] Moldovan JB Moran JV. The Zinc-Finger Antiviral Protein ZAP Inhibits LINE and Alu Retrotransposition. PLoS Genet (2015) 11:e1005121. 10.1371/journal.pgen.1005121 25951186 PMC4423928

[103] Lau L Gray EE Brunette RL Stetson DB. DNA Tumor Virus Oncogenes Antagonize the cGAS-Sting DNA-Sensing Pathway. Science (2015) 350:568–71. 10.1126/science.aab3291 PMC12974531 26405230

[104] Zhao K Du J Peng Y Li P Wang S Wang Y. Line1 Contributes to Autoimmunity Through Both RIG-I- and MDA5-Mediated RNA Sensing Pathways. J Autoimmun (2018) 90:105–15. 10.1016/j.jaut.2018.02.007 29525183

[105] Lander ES Linton LM Birren B Nusbaum C Zody MC Baldwin J. Initial Sequencing and Analysis of the Human Genome. Nature (2001) 409:860–921. 10.1038/35057062 11237011

[106] Brouha B Schustak J Badge RM Lutz-Prigge S Farley AH Moran JV. Hot L1s Account for the Bulk of Retrotransposition in the Human Population. Proc Natl Acad Sci USA (2003) 100:5280–5. 10.1073/pnas.0831042100 PMC154336 12682288

[107] Gao G Guo X Goff SP. Inhibition of Retroviral RNA Production by ZAP, a CCCH-Type Zinc Finger Protein. Science (2002) 297:1703–6. 10.1126/science.1074276 12215647

[108] Zhu Y Chen G Lv F Wang X Ji X Xu Y. Zinc-Finger Antiviral Protein Inhibits HIV-1 Infection by Selectively Targeting Multiply Spliced Viral mRNAs for Degradation. Proc Natl Acad Sci USA (2011) 108:15834–9. 10.1073/pnas.1101676108 PMC3179061 21876179

[109] Burdick R Smith JL Chaipan C Friew Y Chen J Venkatachari NJ. P Body-Associated Protein MOV10 Inhibits HIV-1 Replication at Multiple Stages. J Virol (2010) 84:10241–53. 10.1128/JVI.00585-10 PMC2937795 20668078

[110] Furtak V Mulky A Rawlings SA Kozhaya L Lee K Kewalramani VN. Perturbation of the P-Body Component MOV10 Inhibits HIV-1 Infectivity. PLoS One (2010) 5:e9081. 10.1371/journal.pone.0009081 20140200 PMC2816699

[111] Wang X Han Y Dang Y Fu W Zhou T Ptak RG. Moloney Leukemia Virus 10 (MOV10) Protein Inhibits Retrovirus Replication. J Biol Chem (2010) 285:14346–55. 10.1074/jbc.M110.109314 PMC2863248 20215113

[112] Du J Zhao K. HIV Suppressors Against LINE-1: One Functions as Two. Crit Rev Biochem Mol Biol (2021) 56:205–20. 10.1080/10409238.2021.1893640 33648399

[113] Gao W Li G Bian X Rui Y Zhai C Liu P. Defective Modulation of LINE-1 Retrotransposition by Cancer-Associated SAMHD1 Mutants. Biochem Biophys Res Commun (2019) 519:213–9. 10.1016/j.bbrc.2019.08.155 31492497

[114] Kawano K Doucet AJ Ueno M Kariya R An W Marzetta F. HIV-1 Vpr and P21 Restrict LINE-1 Mobility. Nucleic Acids Res (2018) 46:8454–70. 10.1093/nar/gky688 PMC6144823 30085096

[115] Jones RB Song H Xu Y Garrison KE Buzdin AA Anwar N. LINE-1 Retrotransposable Element DNA Accumulates in HIV-1-Infected Cells. J Virol (2013) 87:13307–20. 10.1128/JVI.02257-13 PMC3838212 24089548

[116] Niewiadomska AM Yu XF. Host Restriction of HIV-1 by APOBEC3 and Viral Evasion Through Vif. Curr Top Microbiol Immunol (2009) 339:1–25. 10.1007/978-3-642-02175-6_1 20012521

[117] Stewart SA Poon B Jowett JB Chen IS. Human Immunodeficiency Virus Type 1 Vpr Induces Apoptosis Following Cell Cycle Arrest. J Virol (1997) 71:5579–92. 10.1128/JVI.71.7.5579-5592.1997 PMC191800 9188632

[118] Goh WC Rogel ME Kinsey CM Michael SF Fultz PN Nowak MA. HIV-1 Vpr Increases Viral Expression by Manipulation of the Cell Cycle: A Mechanism for Selection of Vpr *in vivo* . Nat Med (1998) 4:65–71. 10.1038/nm0198-065 9427608

[119] Levy DN Refaeli Y MacGregor RR Weiner DB. Serum Vpr Regulates Productive Infection and Latency of Human Immunodeficiency Virus Type 1. Proc Natl Acad Sci USA (1994) 91:10873–7. 10.1073/pnas.91.23.10873 PMC45128 7971975

[120] Iijima K Okudaira N Tamura M Doi A Saito Y Shimura M. Viral Protein R of Human Immunodeficiency Virus Type-1 Induces Retrotransposition of Long Interspersed Element-1. Retrovirology (2013) 10:83. 10.1186/1742-4690-10-83 23915234 PMC3751050

[121] Doi A Iijima K Kano S Ishizaka Y. Viral Protein R of HIV Type-1 Induces Retrotransposition and Upregulates Glutamate Synthesis by the Signal Transducer and Activator of Transcription 1 Signaling Pathway. Microbiol Immunol (2015) 59:398–409. 10.1111/1348-0421.12266 25990091

[122] Doehle BP Chang K Fleming L McNevin J Hladik F McElrath MJ. Vpu-Deficient HIV Strains Stimulate Innate Immune Signaling Responses in Target Cells. J Virol (2012) 86:8499–506. 10.1128/JVI.00424-12 PMC3421769 22647704

[123] Banuelos-Sanchez G Sanchez L Benitez-Guijarro M Sanchez-Carnerero V Salvador-Palomeque C Tristan-Ramos P. Synthesis and Characterization of Specific Reverse Transcriptase Inhibitors for Mammalian LINE-1 Retrotransposons. Cell Chem Biol (2019) 26:1095–109.e14. 10.1016/j.chembiol.2019.04.010 31155508

